# Dynamics of T-Lymphocyte Activation Related to Paradoxical Tuberculosis-Associated Immune Reconstitution Inflammatory Syndrome in Persons With Advanced HIV

**DOI:** 10.3389/fimmu.2021.757843

**Published:** 2021-10-07

**Authors:** Rafael Tibúrcio, Beatriz Barreto-Duarte, Gopolan Naredren, Artur T. L. Queiroz, Selvaraj Anbalagan, Kaustuv Nayak, Narayanan Ravichandran, Rajasekaran Subramani, Lis R. V. Antonelli, Kumar Satagopan, Komathi Anbalagan, Brian O. Porter, Alan Sher, Soumya Swaminathan, Irini Sereti, Bruno B. Andrade

**Affiliations:** ^1^ Laboratório de Inflamação e Biomarcadores, Instituto Gonçalo Moniz, Fundação Oswaldo Cruz, Salvador, Brazil; ^2^ Multinational Organization Network Sponsoring Translational and Epidemiological Research (MONSTER) Initiative, Salvador, Brazil; ^3^ Faculdade de Medicina, Universidade Federal da Bahia, Salvador, Brazil; ^4^ Curso de Medicina, Universidade Salvador (UNIFACS), Salvador, Brazil; ^5^ Programa de Pós-Graduação em Clínica Médica, Universidade Federal do Rio de Janeiro, Rio de Janeiro, Brazil; ^6^ Department of Clinical Research, National Institute for Research in Tuberculosis, Chennai, India; ^7^ Center of Data and Knowledge Integration for Health (CIDACS), Instituto Gonçalo Moniz, Fundação Oswaldo Cruz, Salvador, Brazil; ^8^ ICGEB-Emory Vaccine Centre, International Centre for Genetic Engineering and Biotechnology, Aruna Asaf Ali Marg, India; ^9^ Government Hospital of Thoracic Medicine, Chennai, India; ^10^ Laboratório de Biologia e Imunologia de Doenças Infecciosas e Parasitárias, Instituto René Rachou, Fundação Oswaldo Cruz, Belo Horizonte, Brazil; ^11^ HIV Pathogenesis Section, Laboratory of Immunoregulation, National Institute of Allergy and Infectious Diseases, National Institutes of Health, Bethesda, MD, United States; ^12^ Immunobiology Section, Laboratory of Parasitic Diseases, National Institute of Allergy and Infectious Diseases, National Institutes of Health, Bethesda, MD, United States; ^13^ Wellcome Trust Centre for Infectious Disease Research in Africa, Institute of Infectious Disease and Molecular Medicine, University of Cape Town, Cape Town, South Africa; ^14^ Curso de medicina, Escola Bahiana de Medicina e Saúde Pública (EBMSP), Salvador, Brazil; ^15^ Division of Infectious Diseases, Department of Medicine, Vanderbilt University School of Medicine, Nashville, TN, United States

**Keywords:** T lymphocytes, IRIS pathogenesis, TB-HIV coinfection, inflammation, T cell activation

## Abstract

Most persons living with HIV (PLWH) experience a significant restoration of their immunity associated with successful inhibition of viral replication after antiretroviral therapy (ART) initiation. Nevertheless, with the robust quantitative and qualitative restoration of CD4^+^ T-lymphocytes, a fraction of patients co-infected with tuberculosis develop immune reconstitution inflammatory syndrome (TB-IRIS), a dysregulated inflammatory response that can be associated with significant tissue damage. Several studies underscored the role of adaptive immune cells in IRIS pathogenesis, but to what degree T lymphocyte activation contributes to TB-IRIS development remains largely elusive. Here, we sought to dissect the phenotypic landscape of T lymphocyte activation in PLWH coinfected with TB inititating ART, focusing on characterization of the profiles linked to development of TB-IRIS. We confirmed previous observations demonstrating that TB-IRIS individuals display pronounced CD4^+^ lymphopenia prior to ART initiation. Additionally, we found an ART-induced increase in T lymphocyte activation, proliferation and cytotoxicity among TB-IRIS patients. Importantly, we demonstrate that TB-IRIS subjects display higher frequencies of cytotoxic CD8^+^ T lymphocytes which is not affected by ART. Moreover, These patients exhibit higher levels of activated (HLA-DR^+^) and profilerative (Ki-67^+^) CD4^+^ T cells after ART commencenment than their Non-IRIS counterparts. Our network analysis reveal significant negative correlations between Total CD4^+^ T cells counts and the frequencies of Cytotoxic CD8^+^ T cells in our study population which could suggest the existance of compensatory mechanisms for Mtb-infected cells elimination in the face of severe CD4^+^ T cell lymphopenia. We also investigated the correlation between T lymphocyte activation profiles and the abundance of several inflammatory molecules in plasma. We applied unsupervised machine learning techniques to predict and diagnose TB-IRIS before and during ART. Our analyses suggest that CD4^+^ T cell activation markers are good TB-IRIS predictors, whereas the combination of CD4^+^ and CD8^+^ T cells markers are better at diagnosing TB-IRIS patients during IRIS events Overall, our findings contribute to a more refined understanding of immunological mechanisms in TB-IRIS pathogenesis that may assist in new diagnostic tools and more targeted patient management.

## Introduction

The advent of antiretroviral therapy (ART) has substantially reduced the rates of morbidity and mortality associated with HIV infection. With ART, people living with HIV (PLWH) are expected to live longer and healthier lives, as treatment successfully suppresses viral replication and provides partial but substantial restoration of immunity. Although treatment mitigates the development of several opportunistic infections, tuberculosis (TB) remains a serious co-infection that can lead to long-term complications and an increased risk of death (1, 2).

A proportion of PLWH experiences a paradoxical clinical worsening or unmasking of TB within the first few weeks after ART initiation, a phenomenon known as immune reconstitution inflammatory syndrome (IRIS) (3, 4). The reported incidence of TB-related IRIS is associated with TB epidemiological settings and ranges from 8 to 54% (5, 6). Interestingly, factors such as advanced immunological deterioration coupled with high viral loads, short interval between antitubercular treatment (ATT) and ART initiation, as well as the abundance of *Mycobacterium tuberculosis* (Mtb) antigens at the time of immune restoration also play a pivotal role in IRIS pathogenesis (7). Clinical manifestations are largely variable ranging from fever and lymph node enlargement to severe and rapid worsening of respiratory symptoms, and even death (4). Since current TB-IRIS diagnostic tools remain suboptimal, newer accurate methods for early diagnosis and prediction are critical for better patient care and management.

Although TB-IRIS immunopathogenesis is both convoluted and not fully elucidated, a large number of studies suggests the contribution of dysregulated immune activation directed against Mtb antigens in the initiation of systemic inflammation (8–11). Previous reports from our group demonstrated that hyperresponsive activity from both innate and adaptive immune cells characterizes TB-IRIS (8). In fact, a rapid expansion of Mtb-specific CD4^+^ T cells is detected among TB-IRIS patients (10). Notably, Silveira-Mattos et al. recently demonstrated that TB-IRIS individuals display profound alterations in CD4^+^ T lymphocyte memory and effector functions prior to and after treatment (12). Additionally, as observed in previous studies involving IRIS patients with heterogenous co-infections, the frequency of T lymphocyte activation is elevated at pre-ART and is sustained during IRIS occurrence (13, 14).

In the present work, we dissected the immunophenotype of peripheral CD4^+^ and CD8^+^ T cells in a cohort of HIV-1 infected individuals diagnosed with pulmonary TB (PTB) before and after ART commencement. Our data showed that patients at higher risk of developing TB-IRIS exhibited more severe CD4^+^ T cell lymphopenia then those who did not. Similarly, TB-IRIS subjects showed a higher degree of CD4^+^ T lymphocyte activation, proliferation, and exhaustion dynamics over time with ART. Notably, these patients also displayed early and sustained expression of CD8^+^ T cell cytotoxicity markers. Furthermore, hyperactivated T lymphocytes from IRIS patients exhibited strong positive correlations with plasma concentrations of several inflammatory biomarkers before and after ART initiation. Finally, we employed a multidimensional integrative analytical model combining T cell activation-related markers to predict and/or diagnose TB-IRIS. Collectively, the data shown in our study suggest that the patterns of T lymphocyte activation can aid in distinguishing TB-IRIS subjects from their non-IRIS counterparts before and during the IRIS occurrence.

## Methods

### Ethics Statement

All clinical investigations were carried out in accordance with the principles disclosed in the Declaration of Helsinki. Of note, the pre-enrollment stage involved obtaining of written informed consent from all participants of the present investigation. This study was approved by the Scientific Advisory Committee and Institutional Ethics Committee of the National Institute for Research in Tuberculosis (Chennai) and registered on Clinicaltrials.gov (NCT00933790).

### Description of Study Population

The present study is a retrospective analysis of data collected from a previously published cohort (6). The Indian TB-IRIS cohort study comprised an observational analysis nested within a randomized controlled trial (NCT00- 933790) at the National Institute for Research in Tuberculosis (NIRT) in Chennai, India. In this retrospective investigation, HIV-1 infected patients with recent diagnosis of sputum culture-confirmed pulmonary TB were enrolled as previously reported (6). The parent randomized controlled clinical trial primarily compared outcomes of daily versus intermittent anti-TB regimens in the aforementioned group of individuals (15). Eligibility criteria were based on patient age (above 18 years old), with rifampicin-sensitive TB, and ART-naïve status. Clinical evaluation and blood samples were collected at pre-ART (baseline) and at the time of IRIS occurrence or equivalent time point (usually between 2 to 6 weeks following ART initiation). IRIS was confirmed by an independent panel of experts who were presented with the de-identified data of patients after the exclusion of drug resistance, and ruling out of endemic infections, with a thorough scrutiny for febrile episodes. IRIS was diagnosed by following a modified International Network for the study of HIV-associated IRIS (INSHI) guideline. This took into consideration both expected decreases in HIV plasma viral load (of at least 0.5 log) and sputum to assess Acid-Fast Bacillus (AFB)-negativity by culture or a decline in grade. All patients exhibited increases in CD4^+^ T cell counts concomitantly with dramatically reduced plasma viral load. The detailed clinical, laboratory, and microbiologic description of the study participants has been previously reported (6).

### Measurement of Plasma Biomarkers

Concentrations of C-reactive protein (CRP) (eBioscience, San Diego, CA), Eotaxin, Fibroblast growth factor (FGF) – basic, FMS-like tyrosine kinase 3 ligand (FLT3L), Granulocyte colony-stimulating factor (G-CSF), intestinal fatty acid binding protein (I-FABP) (Hycult Biotech, The Netherlands), Interferon (IFN) -α, IFN-β, IFN-γ, Interleukin (IL)- 1Ra, IL-1β, IL-2, IL-4, IL-5, IL-6, IL-7, IL-8, IL-9, IL-10, IL-12p40, IL-12p70, IL-13, IL-15, IL-17, Interferon-γ-induced protein 10 (IP-10/CXCL10), Monocyte chemoattractant protein-1 (MCP-1/CCL2), Macrophage inflammatory protein (MIP-1α/CCL3), MIP-1β (CCL4), Platelet-derived growth factor (PDGF), Regulated on activation normal T cell expressed and secreted (RANTES/CCL5), soluble CD14 (sCD14), soluble CD163 (sCD163), soluble Granzyme B (sGzB), soluble Programmed Cell death protein (PD) -1, soluble Tissue factor (sTF), Transforming Growth Factor (TGF)-β, Tumor Necrosis Factor (TNF)-α, and vascular endothelial growth factor (VEGF) (Bio-Plex, Bio-Rad, Hercules, CA)were assessed in cryopreserved plasma samples maintained at −80 °C.

### Cell Staining and Flow Cytometry Assay

In order to dissect the immunophenotype of T lymphocytes, we evaluated markers associated with activation (HLA-DR), exhaustion (PD-1), proliferation (ki-67), and cytotoxicity (Granzyme B [GzB]). Briefly, this characterization was conducted by staining aliquots of 250 µL of whole blood with the following antibodies: CD3, CD4, CD8, HLA-DR, PD-1, Ki-67, and GzB. All antibodies were obtained from eBioscience (San Diego, CA), Biolegend (San Diego, CA), BD Biosciences (San Jose, CA) and Life Technologies (Carlsbad, CA). The antibody panel was prepared in PBS 1% BSA for 30 minutes at room temperature. Data were acquired on a BD FACS Canto II flow cytometer (BD Biosciences). All compensation and gate analysis were conducted in FlowJo 9.5.3 (TreeStar, Ashland, OR).

### Network Analysis

The inferential networks were generated from Spearman correlation matrices containing values of each biomarker measured in the plasma samples and flow cytometry markers of T cell activation. All values were inputted and analyzed in *circusplot* R package. The links shown in the networks represent statistically significant Spearman rank correlations (P<0.05). Additionally, we dissected the structure of networks by calculating the network density. The density measure is defined as follows: density = L/(N (N-1)/2), in which L is the number of observed edges (i.e., Spearman correlations with P<0.05) and N is the total number of the nodes in the network. The density is normalized, ranging between 0 (no edges in the network) and 1 (all possible edges presents). Graphics for the network analysis were customized using *circosplot* R package and Adobe Illustrator (Adobe Systems Inc.).

### Data Analysis

Median values with IQR or frequencies of variables were compared using the Mann-Whitney *U* test (when two groups were compared) or the Kruskal-Wallis test with Dunn’s multiple comparisons *ad hoc* analysis (when three groups were compared). Fisher’s exact test or Chi-square tests were used to compare two or three groups, respectively, for proportions. Paired changes from before ART initiation to week 6 or the time of IRIS development were compared using the Wilcoxon matched-paired T test. Using JMP 10.0 software, geometric mean values (log_10_) for each marker measured at week 0 and week 6 were calculated for the entire study population. To assess the overall pattern of expression of these markers in each clinical group and timepoint, heatmaps were built using variation from the geometric mean value calculated for each candidate biomarker. Principal component analysis was performed using the *Factorextra* R package. A hierarchical cluster analysis using the Ward’s method was employed to reveal patterns of expression in plasma. Receiver operator Characteristic (ROC) curve analysis was performed on pROC package (16). Throughout the text, a p-value of <0.05 was considered statistically significant after adjustments for multiple measurements (Holm-Bonferroni’s correction method). The statistical analyses were performed using GraphPad Prism 9.0 (GraphPad Software Inc., USA), the ggplot2 R package (version 3.10), and STATA 9.0 (StataCorp, TX, USA).

## Results

### TB-IRIS Patients Display Altered CD4^+^ and CD8^+^ T Lymphocyte Frequencies Compared to Their Non-IRIS Counterparts

**Figure 1:**
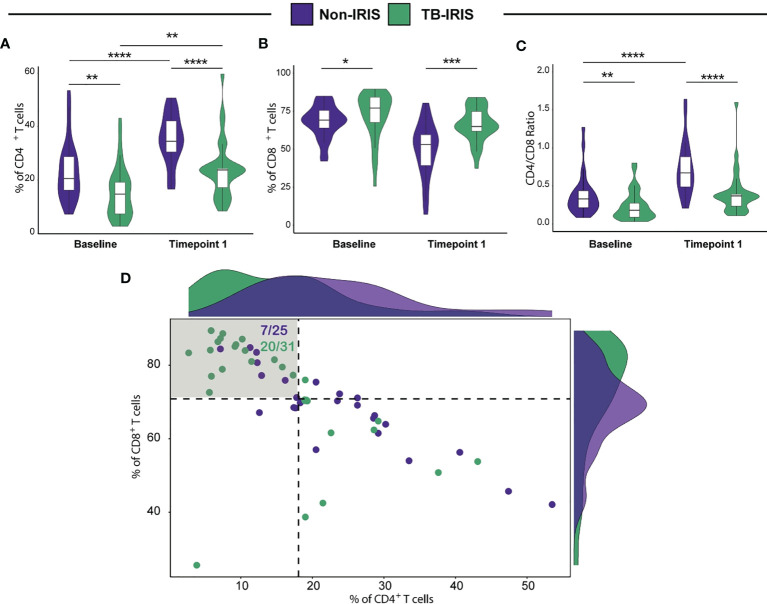
Assessment of both CD4^+^ and CD8^+^ T cell frequencies in HIV-infected individuals prior to antiretroviral therapy (ART) initiation and during TB-IRIS manifestations. Comparison of CD4^+^ **(A)** and CD8^+^ **(B)** T cell frequencies in patients who developed TB-IRIS and those who did not at ART initiation (Baseline) and at the time of TB-IRIS manifestations (Timepoint 1), which occurred between 2 and 6 weeks after ART initiation. **(C)** CD4/CD8 T cell ratios at the aforementioned conditions. **(D)** Scatterplot displaying CD4^+^ and CD8^+^ T cell frequencies prior to ART initiation. Patients who developed TB-IRIS are depicted by green dots whereas those who did not experience TB-IRIS manifestations are represented by purple dots. Dotted lines on the X and Y axes represent the median value of CD4^+^ and CD8^+^ T cell counts within the entire study population, respectively. The shadowed quadrant represents those individuals with CD8^+^ T cell counts above the median value and CD4^+^ T cell frequencies below the median value for the entire population. Data were analyzed using the Mann-Whitney test or Wilcoxon matched-pairs test for paired analyses within each study group (*p < 0.05, **p < 0.01, ***p < 0.001, ****p < 0.0001).

A total of 56 HIV^+^ patients who were recently diagnosed with pulmonary TB and who were both ATT and ART-naïve were enrolled in this study. The baseline description of patients enrolled in study is presented in **Supplementary Table 1**. As demonstrated by previous studies of our group, several factors contribute to the risk of IRIS development, including low CD4^+^ T lymphocyte counts prior to ART initiation. Therefore, we devised a flow cytometric approach to dissect the phenotype landscape of T lymphocytes in our study population at pre-ART and at 2-6 weeks after treatment initiation. We observed that baseline CD4^+^ T cell frequencies among CD3^+^ lymphocytes were significantly lower among TB-IRIS patients when compared to Non-IRIS individuals. Similarly, at 2-6 weeks post-treatment initiation, the amounts of CD4^+^ T lymphocytes remained lower in the TB-IRIS group (**Figure 1A**). Of note, as an expected effect of ART, both groups displayed higher percentages of CD4^+^ T cells at 2-6 weeks following treatment initiation when compared to baseline (**Figure 1A**). Interestingly, we also detected significantly higher CD8^+^ T cell percentages in TB-IRIS subjects both at ART initiation and during 2-6 weeks after the start of treatment (**Figure 1B**). Next, we sought to determine the CD4^+^/CD8^+^ T lymphocyte ratio at both week 0 and 2-6 weeks after ART. Our analysis revealed that Non-IRIS individuals displayed higher CD4^+^/CD8^+^ T lymphocyte ratio when compared to their IRIS counterparts at both studied time points (**Figure 1C**). Additionally, we employed a combined measurement of both CD4^+^ and CD8^+^ T cell frequencies at ART initiation to further distinguish TB-IRIS from Non-IRIS individuals. Thus, we found that, when compared to the median value of the overall population, approximately 65% of TB-IRIS individuals displayed higher CD8^+^ and lower CD4^+^ T cell frequencies (**Figure 1D**). These results reinforce the importance of severe CD4^+^ lymphopenia in IRIS pathogenesis, highlighting a unique profile of CD4/CD8 ratio.

### Anti-Retroviral Treatment Alters Immunopathological Responses to *Mycobacterium tuberculosis*

**Figure 2:**
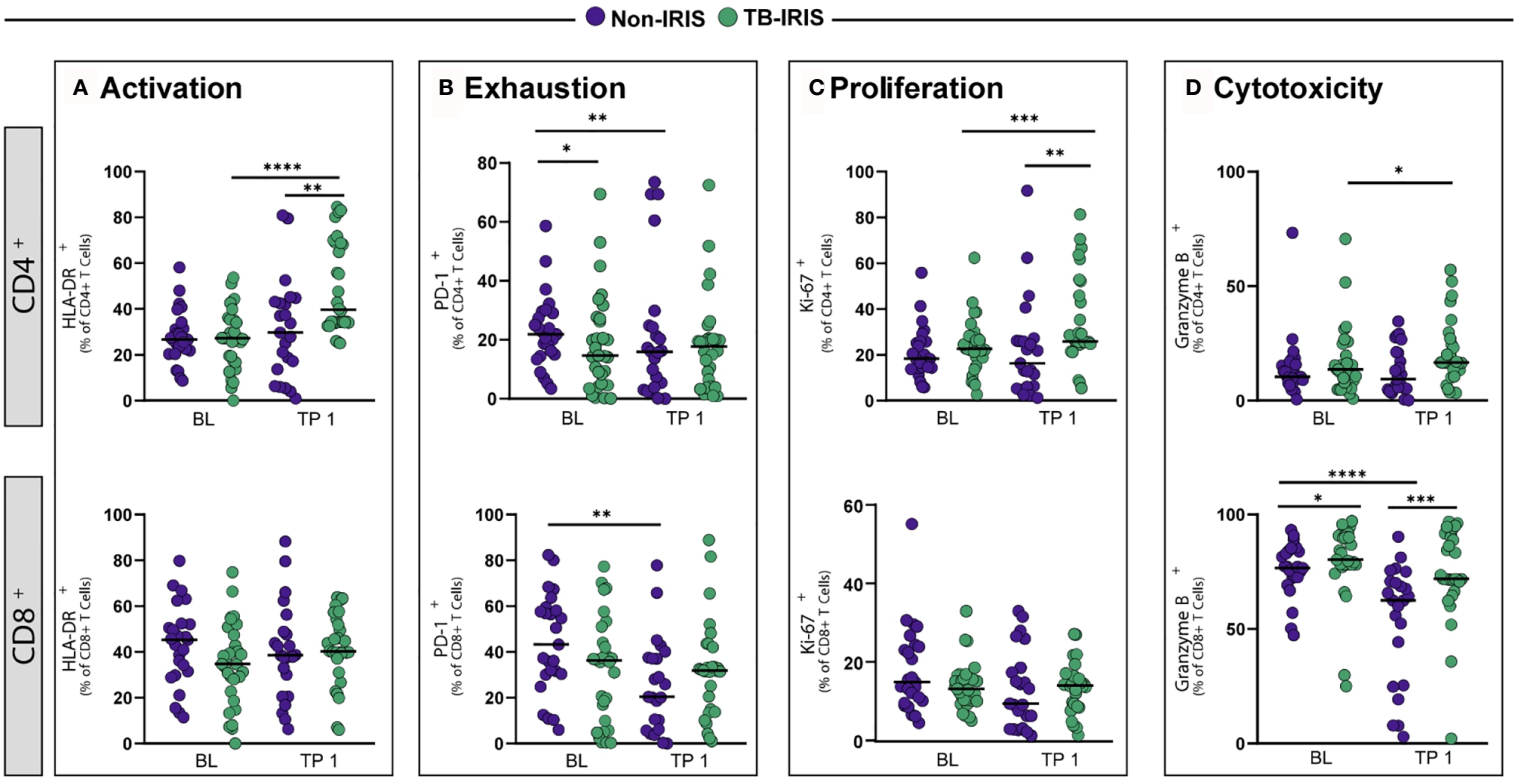
Evaluation of T lymphocyte activation markers in TB-HIV co-infected patients prior to and following ART initiation. Expression of flow cytometry markers related to T cell activation (HLA-DR) **(A)**, exhaustion (PD-1) **(B)**, proliferation (Ki67) **(C)**, and cytotoxicity (Granzyme B) **(D)** were assessed in whole blood samples from TB-HIV co-infected individuals at baseline (BL) pre-ART and at the time of TB-IRIS manifestation (Timepoint [TP]1), which was 2-6 weeks on ART. Data were analyzed using the Mann-Whitney test or Wilcoxon matched-pairs test for paired analyses within each study group (*p < 0.05, **p < 0.01, ***p < 0.001, ****p < 0.0001).

Next, we wished to evaluate the impact of ART on T lymphocyte activation in TB-IRIS and Non-IRIS patients. We observed that the frequencies of activated CD4^+^ T cells (measured as the percentage of HLA-DR^+^ T cells) were similar in both groups prior to ART; however, TB-IRIS individuals displayed augmented levels of activated CD4^+^ T cells at 2-6 weeks period in comparison to the Non-IRIS group. Additionally, we found that TB-IRIS persons exhibited higher frequencies of activated CD4^+^ lymphocytes after ART initiation when compared to baseline levels. Interestingly, the percentages of HLA-DR^+^ CD8^+^ T cells were similar in the overall population regardless of ART initiation (**Figure 2A**). We then extended these findings on T cell activation to explore the effects of treatment on other characteristics of T cells such as exhaustion (PD-1^+^ cells), proliferation (ki-67^+^ cells), and cytotoxicity (Granzyme B^+^ [GrB^+^] Cells). We noticed that Non-IRIS persons had higher baseline levels of both exhausted CD4^+^ and CD8^+^ T cells, and that ART reduced substantially the amount of PD-1^+^ T cells. Additionally, at ART initiation, the percentage of PD1^+^CD4^+^ cells was higher among Non-IRIS compared to TB-IRIS individuals (**Figure 2B**). We also verified that TB-IRIS persons displayed higher amounts of proliferative CD4^+^ T cells (measured as the percentage of Ki67^+^ cells) in comparison to the Non-IRIS group at 2-6 weeks post-ART initiation. Of note, we did not detect any significant difference in CD8^+^ T cell proliferation status between these two groups (**Figure 2C**). Regarding cytotoxicity, we found higher frequencies of GrB^+^ CD8^+^ T cells in TB-IRIS patients, and such levels remained elevated even after ART commencement. On the other hand, we were able to detect a pronounced decline in GrB^+^ CD8^+^ levels of Non-IRIS individuals after ART. Interestingly, TB-IRIS individuals had augmented frequencies of GrB^+^ CD4^+^ at weeks 2-6 (**Figure 2D**). Our data show that TB-IRIS patients display heightened T lymphocyte activation with increased frequencies of proliferative CD4^+^ and cytotoxic CD8^+^ T cells during IRIS.

### Network Analysis in TB-IRIS and Non-IRIS HIV-Infected Persons Reveals Nuances in T Lymphocyte Activation Patterns

**Figure 3:**
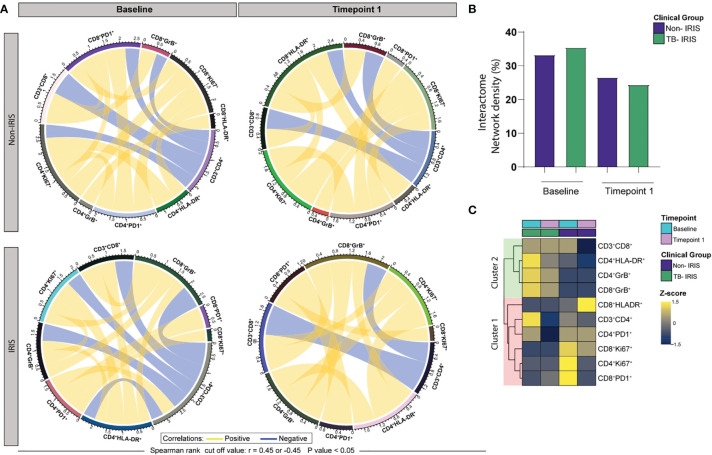
ART initiation and TB-IRIS events are associated with coordinated alterations in the relationship between markers of T lymphocyte activation at Baseline (pre-ART) and 2-6 weeks following ART initiation. **(A)** Network analysis of T cell populations expressing activation-related markers; correlation matrices were developed with bootstrap (100 ×). **(B)** Networks depicting only significant correlations (p < 0.05), with Spearman rank threshold ± 0.5; graph showing interactome network density among the clinical groups. **(C)** A heatmap was employed to assess the contribution of each node based on the number of statistically significant correlations; yellow lines (edges) represent positive correlations between nodes (T cell population expressing activation-related markers), whereas blue lines depict negative correlations.

We next conducted Spearman correlation-based network analysis to investigate how ART initiation and IRIS development associate with T lymphocyte activation. By employing circular layout networks to depict correlations of T cell activation markers, we observed that both IRIS and Non-IRIS patients exhibited similar correlation patterns at the pre-treatment timepoint and after ART initiation (**Figure 3A**). Nevertheless, our interactome analysis revealed that IRIS subjects displayed higher median network density (ND) before treatment and lower ND at the 2–6-week period when compared to non-IRIS patients (**Figure 3B**). Additionally, we performed node analysis of all T cell activation-related markers that displayed statistically significant correlations in both groups. Of note, our analysis revealed that CD4^+^ T lymphocytes (HLA-DR^+^ cells) and cytotoxic CD8^+^ T cells figured as top node among IRIS patients, while exhausted CD8^+^ and proliferative CD4^+^ T lymphocytes exhibited the highest number of correlations in the Non-IRIS group prior to ART. After 2-6 weeks of treatment, cytotoxic CD8^+^ T cells were top nodes in the IRIS group, whereas among Non-IRIS patients, activated CD4^+^ T lymphocytes showed the largest number of correlations (**Figure 3C**). These results argue that the identification of T lymphocyte patterns of activation may contribute to a better understanding of the immunological nuances surrounding IRIS.

### T Lymphocyte Activation Correlates With Distinct Profiles of Systemic Inflammation Between TB-IRIS and Non-IRIS Patients

**Figure 4:**
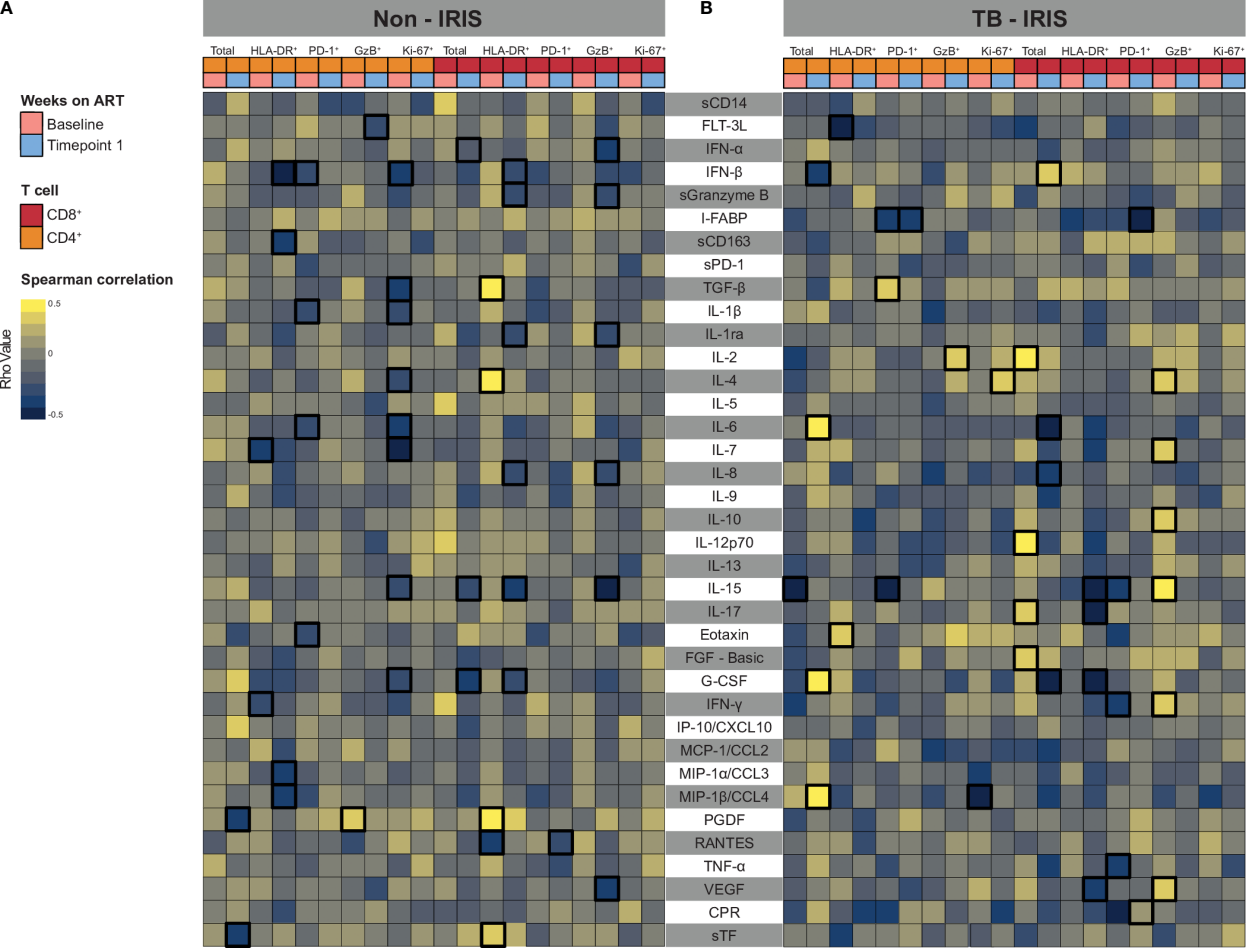
Associations between both CD4+ and CD8+ T lymphocytes expressing activation-related biomarkers and plasma levels of several inflammatory biomarkers. A heatmap was employed to depict the strength of association between CD4+ and CD8+ T cells expressing activation-related biomarkers and the level of circulating cytokines and chemokines at Baseline (prior to ART) and after approximately 2 to 6 weeks following ART initiation (Timepoint 1) for Non-IRIS patients **(A)** and individuals who experienced TB-IRIS manifestations **(B)**. Statistically significant correlations (P < 0.05) after adjustment for multiple measurements are highlighted with bold squares.

Considering that activated T lymphocytes play a role in systemic inflammation, we investigated how ART and IRIS development could influence the correlation between T cell activation markers and plasma levels of several inflammation-related molecules (cytokines, chemokines, and growth factors). Overall, Non-IRIS patients exhibited early and sustained negative correlations between activation markers and inflammatory molecule levels. In this group, activated CD4^+^ T lymphocytes were negatively correlated with IL-7 as well as IFN-γ prior to treatment, and with IFN-β, sCD163, MIP1-α, and MIP1-β 2-6 weeks later. On the other hand, HLA-DR^+^CD8^+^ T cells displayed positive correlations with TGF- β, IL-4, and PDGF, while negatively associated with RANTES at the baseline timepoint. At 2-6 weeks post-treatment, these lymphocytes showed negative correlations with several markers (IFN-β, soluble Granzyme B (sGrB), Il-1Ra, IL-8, IL-15, G-CSF) (**Figure 4A**). Concerning IRIS patients, we could detect a larger number of positive correlations of markers of T cell activation with the measured inflammatory molecules. Of note, we found that activated CD4^+^ T cells negatively correlate with FLT-3L and positively with FGF-basic only at the pre-treatment timepoint. Interestingly, activated CD8^+^ T cells were negatively associated with IL-15, IL-17, IFN-γ, and VEGF after treatment initiation (**Figure 4B**). Among ART-naive Non-IRIS individuals, exhausted CD4^+^ T cells were negatively correlated with IFN-β, IL-1β, IL-6, and eotaxin, whereas proliferative CD4^+^ T cells negatively associated with IFN-β, TGF-β, IL-1β, IL-4, IL-6, IL-7, IL-15, and G-CSF. Interestingly, after 2-6 weeks of treatment, exhausted CD8^+^ T cells correlated negatively only with RANTES, whereas cytotoxic lymphocytes with several molecules (IFN-α, sGrB, IL-1Ra, IL-8, IL-15, and VEGF). Among IRIS patients, we observed that exhausted CD4^+^ T lymphocytes were negatively correlated with and the Intestinal Fatty acid-binding protein(I-FABP) before and after ART initiation. Conversely, we found that these lymphocytes displayed positive correlations with TGF-β, and IL-15 only in the baseline timepoint. Of note, we detected early positive correlations between cytotoxic CD8^+^ T lymphocytes and IL-4, IL-7, IL-10, IL-15, IFN-γ, and VEGF. Altogether, these finding highlight how differential T cell activation in IRIS and Non-IRIS groups play a role in systemic inflammation upon ART initiation and IRIS pathogenesis.

### Combination Models of CD4^+^ and CD8^+^ T Cell Activation Markers Distinguish TB-IRIS From Non-IRIS Patients

**Figure 5:**
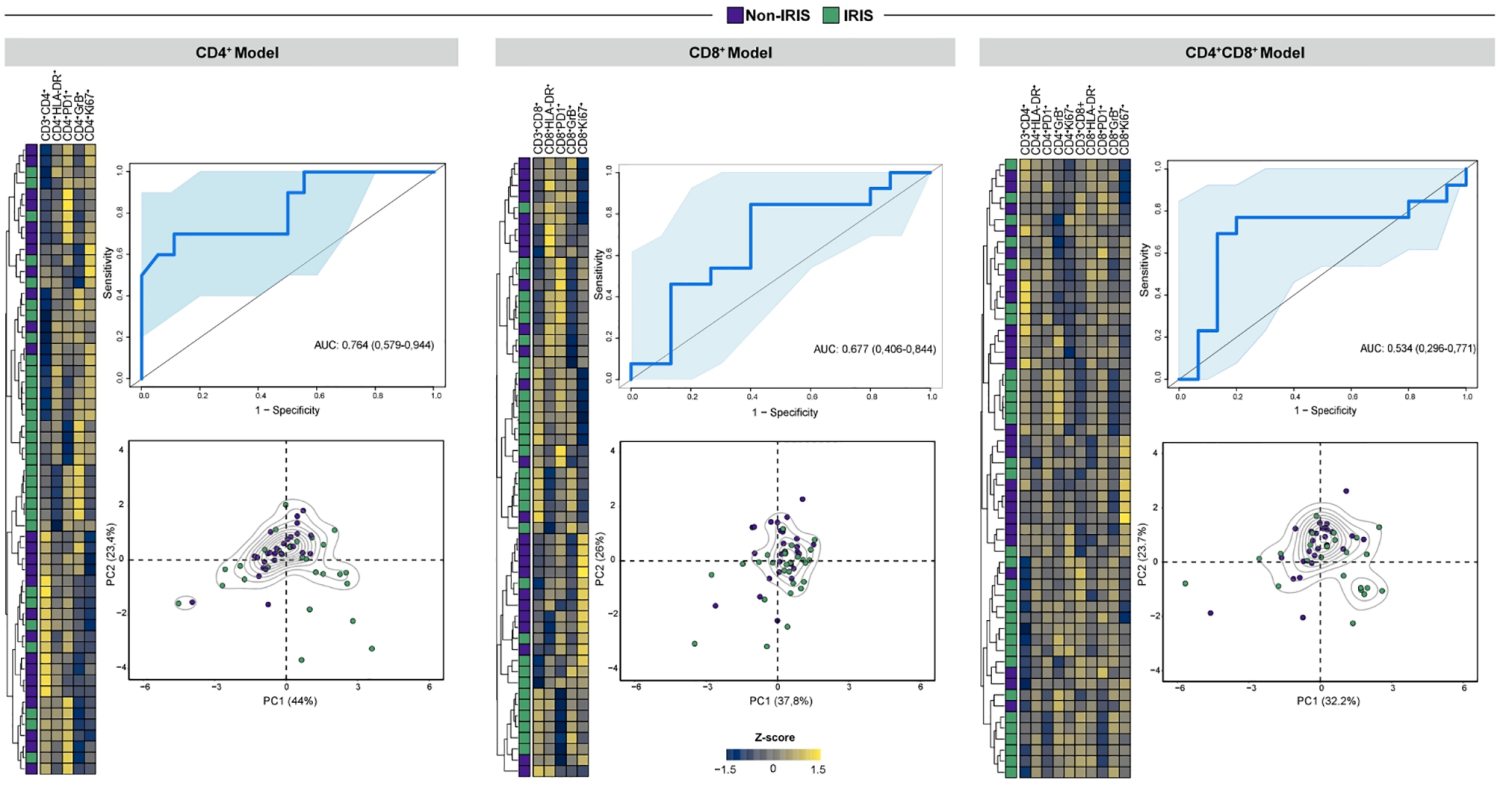
Assessment of CD4+ and CD8+ T lymphocyte activation markers to predict TB-IRIS prior to ART. In order to create predictive models that distinguish TB-IRIS from non-IRIS patients prior to ART, unsupervised machine learning techniques were employed for either CD4^+^ or CD8^+^ T cell activation markers. In addition, a model encompassing both CD4^+^ and CD8^+^ T cell activation markers was also created. For each model, unsupervised cluster analyses (Ward’s method) and principal component analyses were employed. In order to estimate the accuracy of these models in discriminating patients, a receiver-operating characteristic curve analysis was employed.

**Figure 6:**
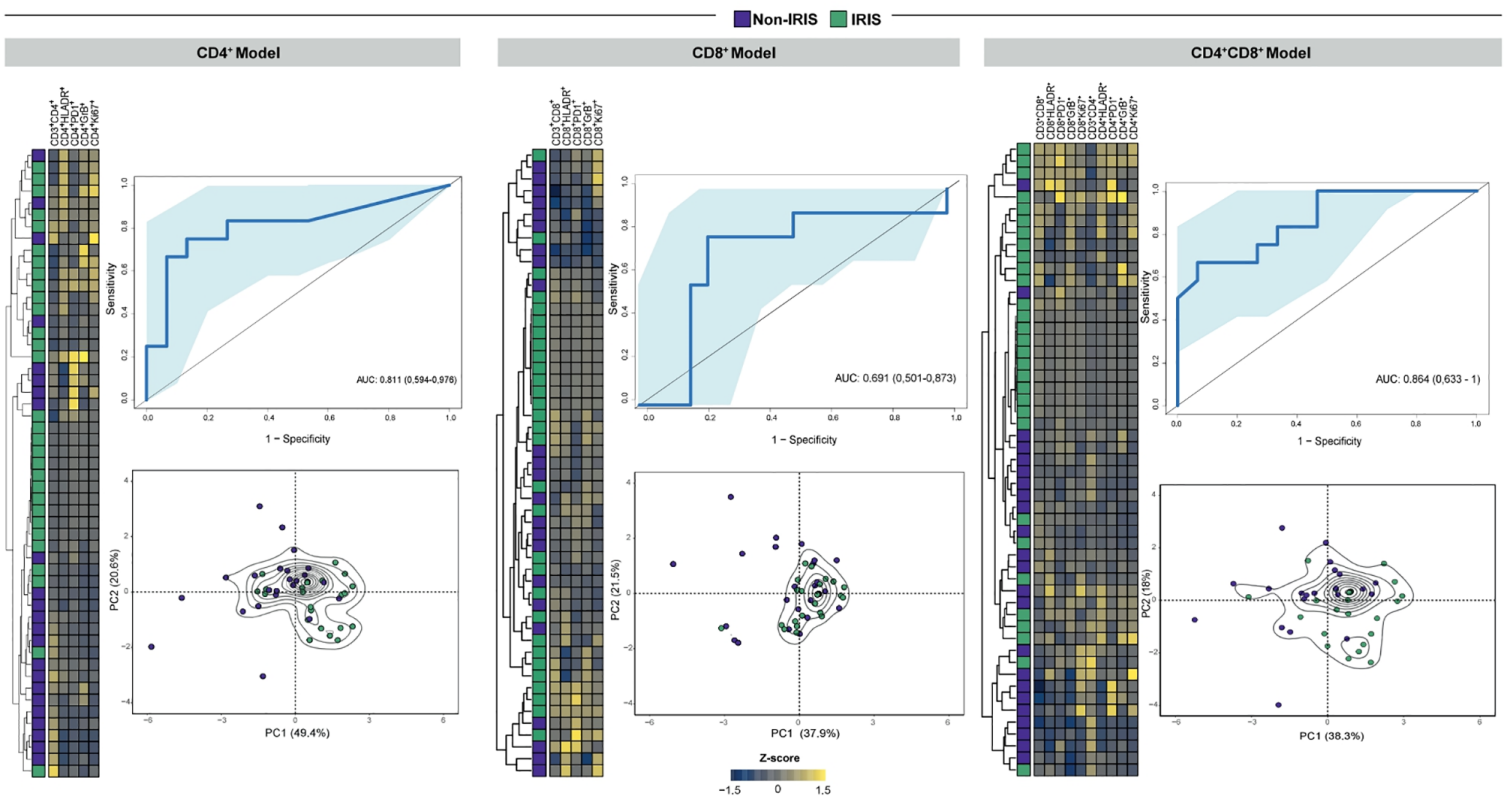
Combination of T cell activation markers to diagnose IRIS after ART initiation. In order to create diagnostic models that distinguish TB-IRIS from non-IRIS patients after ART initiation, unsupervised machine learning techniques were employed for either CD4^+^ or CD8^+^ T cell activation markers. In addition, a model encompassing both CD4^+^ and CD8^+^ T cell activation markers was also created. For each model, unsupervised cluster analyses (Ward’s method) and principal component analyses were employed. In order to estimate the accuracy of these models in discriminating patients, a receiver-operating characteristic curve analysis was employed.

Bearing in mind the role of adaptive immunity hyperactivation in TB- IRIS pathogenesis, we aimed to estimate the predictive and diagnostic value of T cell activation-related markers in our patient cohort. Therefore, we employed unsupervised machine learning techniques with different combinations of flow cytometry markers of CD4^+^ and CD8^+^ T cell activation to build models with predictive and diagnostic potential. Considering T cell markers at the timepoint prior to ART initiation, our first predictive model (only composed of CD4^+^ T cell activation markers) was better than our second (with only CD8^+^ T cell activation markers) and third (incorporating both CD4^+^ and CD8^+^ T cell activation markers) models (**Figure 5**). This result further suggests that activated CD4^+^ T cells contribute more substantially to IRIS prediction than CD8^+^ T cells. Of note, early diagnosis of TB-IRIS is critical to patient management. Thus, we employed our combinatory models considering only T cell activation dynamics after 2 to 6 weeks after ART commencement. Our first diagnostic model also displayed superior capacity to discriminate IRIS from Non-IRIS patients (AUC of 0.811 vs. 0.691). Interestingly, our third diagnostic model showed increased power of discriminating patients (AUC: 0.864) (**Figure 6**). Collectively, our results indicate CD4^+^ T cell activation markers can serve as potential predictors of TB-IRIS, while the combination of CD4^+^ and CD8^+^ T cell markers is better at diagnosing TB-IRIS patients experiencing IRIS.

## Discussion

In the present work, we sought to evaluate the patterns of T lymphocyte activation associated with TB-IRIS development following the start of ART in PLWH displaying high CD4^+^ T lymphocyte suppression pre-ART. Our phenotypic characterization of T cells revealed that TB-IRIS patients exhibited lower CD4^+^ and higher CD8^+^ T lymphocyte counts before ART in comparison to Non-IRIS individuals. These results are consistent with previous work that suggested severe CD4^+^ T cell lymphopenia prior to ART initiation is a risk factor for IRIS development (14, 17). To better understand the role of T lymphocyte activation in IRIS, we performed immune profiling of both circulating CD4^+^ and CD8^+^ T cells regarding expression of activation (HLA-DR), exhaustion (PD-1), proliferation (Ki-67), and cytotoxicity (Granzyme B) markers.

Our working hypothesis was that aberrant T lymphocyte activation plays a pivotal role in exacerbating systemic inflammation as seen in TB-IRIS. We showed that TB-IRIS patients displayed higher levels of CD4^+^ T cell activation at 2-6 weeks after ART initiation than their Non-IRIS counterparts. These findings reiterate those of Antonelli et al. that investigated a US cohort of patients with fungal, bacterial, and viral co-infections (14). Interestingly, a cross-sectional study conducted by Sangwan et al. revealed a correlation between the expression of immune activation markers and disease stage, suggesting that CD8^+^CD38^+^ T cells are associated with viral load in individuals with virological failure (18). Another striking observation of our study was the identification of enhanced levels of both exhausted and proliferative CD4^+^ T cells, as well as Granzyme B^+^CD8^+^ T lymphocytes in TB-IRIS subjects. Of note, by studying a cohort of non-immunological and immunological responders, Piconi et al. showed that patients with a lower nadir CD4^+^ exhibited inferior expansion of a Ki67^+^CD4^+^ T cell population after 7 years of ART, which was attributed to T cell hyperactivation (19). Along these lines, it is feasible that during a TB-IRIS event, patients experience an overwhelming CD4^+^ T cell turnover (as evidenced by higher frequencies of proliferation and exhaustion markers) driven by excessive immune activation elicited by underlying TB infection. A study conducted by Massanella et al. provides compelling evidence for the association between CD4^+^ T cell hyperactivation and increased cell turnover, culminating in cell death (20). Regarding the sustained high frequencies of cytotoxic CD8^+^ T cells detected in TB-IRIS patients; we hypothesize that this might be a compensatory mechanism for the elimination of mycobacterium-infected cells in the face of substantial CD4 lymphopenia. Additionally, our data suggest that the sustained lower levels of activated CD4^+^ T cells along with ART-induced decreases in cellular exhaustion indicate a more robust restoration of T lymphocyte homeostasis in Non-IRIS patients. Thus, our study extends the understanding of the diverse patterns of cellular activation and that CD4^+^ T cells may exert a predominant role in TB-IRIS pathophysiology.

We also dissected T lymphocyte dynamics of activation in TB-IRIS patients by employing network analysis. Interestingly, we found lower values of network density in TB-IRIS patients before ART commencement, followed by a surprising increase in node connectivity during the IRIS event. In the pre-treatment timepoint, HLA-DR^+^CD4^+^ T cells, along with cytotoxic CD8^+^ T cells, exhibited the highest numbers of significant correlations among IRIS patients. Of note, GrB^+^CD8^+^ T lymphocytes remained among the top nodes after 2-6 weeks on ART. A previous study from Wilkinson et al. underscored a substantial role of cytotoxic mediators in TB-IRIS immunopathogenesis (21). Similarly, Hsu et al. observed a role for the expansion of cytotoxic CD4^+^ T cells in Mycobacterium avium complex-IRIS (22). In our analysis, exhausted CD8^+^ and proliferative Ki67^+^ CD4^+^ T cells seemed to be central elements in network density among Non-IRIS subjects. Additionally, we aimed to investigate how differential dynamics of T cell activation were correlated with plasma markers of systemic inflammation. In the Non-IRIS group, we observed that both activated (HLA-DR^+^) CD4^+^ and CD8^+^ T cells were negatively correlated with several inflammation-related molecules before and after ART initiation. Interestingly, in this same group of patients, we observed that HLA-DR^+^ CD8^+^ T cells were negatively correlated with IFN-β, sGZB, IL-8, IL-15, and G-CSF. These negative correlations could be part of a wider phenomenon of mycobacterium containment aided by activated CD8^+^ T lymphocyte in parallel with a more coordinated mobilization of inflammatory molecules resulting in limited immunopathology. Conversely, in TB-IRIS patients, activated CD4^+^ T cells displayed a substantially lower number of statistically significant correlations with plasma molecules, whereas cytotoxic CD8^+^ T lymphocytes were positively associated with plasma levels of IL-4, IL-7, IL-10, IL-15, IFN-γ, and VEGF prior to ART. These findings argue that heightened responses of cytotoxic CD8^+^ T lymphocytes could trigger cell death-related signaling pathways in infected macrophages which could eventually culminate in both amplification of inflammatory responses and exacerbated pathology. Further studies are warranted to more precisely investigate the role of cytotoxic CD8^+^ T cells in the settings of TB-IRIS immunopathogenesis. Of interest, a previous study from our group demonstrated a direct association between frequency of effector CD4^+^ T cell subpopulations and the circulating levels of inflammatory cytokines, further suggesting the participation of T cell immune activation in augmented systemic inflammation detected in TB-IRIS (12). Collectively, these findings lend support to the notion that immune activation-driven systemic inflammation is indeed crucial to promote TB-IRIS pathogenesis.”s

Notably, the overwhelming complexity of TB-IRIS immunopathogenesis impedes the attainment of accurate predictive and diagnostic methods, thus hindering appropriate patient care and disease management. Currently, a TB-IRIS diagnosis is mainly based on clinical presentation and the initial response of the underlying TB infection to ATT, with a paradoxical deterioration after ART initiation determine from both clinical assessment and imaging methods (such as chest X ray and computed tomography) (23). Therefore, major efforts have been devoted to the identification of prediction and diagnostic biomarkers to enhance TB-IRIS management strategies. In a previous retrospective study, we observed that increased baseline levels of monocyte and Th1 cell activation, alongside inflammatory markers, were independently associated with the risk of IRIS development (24). This prompted us to investigate the potential of T cell activation markers as predictive and diagnostic tools for TB-IRIS. By creating models of T cell activation patterns, we found that markers of CD4^+^ T cell activation are better at predicting the occurrence of IRIS in our cohort. Additionally, combined CD4 and CD8 T cell activation markers were more accurate at distinguishing TB-IRIS patients from their Non-IRIS counterparts. To the best of our knowledge, this is the first work to employ flow cytometry markers of T cell activation as a possible tool for TB-IRIS diagnosis.

The main limitation of this study is the small number of patients included in our cohort. Nevertheless, the strength of the current investigation was the selection of a homogeneous cohort of individuals who were naïve to both ART and ATT and presented positive bacillar cultures, with drug-sensitive *Mycobacterium tuberculosis* strains. Moreover, the blood draws taken at the time of IRIS, before administering a single dose of anti-inflammatory medication, enhances our validity. Additionally, patients were carefully monitored since ART initiation for the identification of IRIS events, with immediate collection of blood samples at the first suspicion of an event. Collectively, our findings provide new insights to decipher fundamental events in the immune activation landscape surrounding TB-IRIS pathophysiology.

## Data Availability Statement

The raw data supporting the conclusions of this article will be made available by the authors, without undue reservation.

## Ethics Statement

The studies involving human participants were reviewed and approved by Institutional Ethics Committee of the National Institute for Research in Tuberculosis (Chennai) and registered on Clinicaltrials.gov (NCT00933790). The patients/participants provided their written informed consent to participate in this study.

## Author Contributions

BA, IS, SS, BP, and AS conceptualized the study. BA and IS supervised the immunological study. GN and SS supervised the clinical study. IS, SS, and AS acquired funding for research. GN, RS, KS, BP, IS, and BA performed the clinical assessments. SA, KN, KA BA, and LA performed the experiments. RT, BB-D, and AQ analyzed the data. RT, BB-D, and BA drafted the first version of the manuscript. All the authors revised the manuscript. All authors contributed to the article and approved the submitted version.

## Funding

This work received supported from the Intramural Research Program of National Institute of Allergy and Infectious Diseaes (NIAID/NIH) and by the Intramural-to-India grant from the US-India Co-operative research program. This study was also financed in part by Coordenação de Aperfeiçoamento de Pessoal de Nível Superior (CAPES) (Finance Code 001). The work of BA is supported by the Intramural Research Program of the Oswaldo Cruz Foundation (FIOCRUZ) and the National Council for Scientific and Technological Development (CNPq), Brazil. RT and BB-D were supported by PhD fellowships from CAPES. The funders had no role in study design, data collection and analysis, decision to publish, or preparation of the manuscript.

## Conflict of Interest

The authors declare that the research was conducted in the absence of any commercial or financial relationships that could be construed as a potential conflict of interest.

## Publisher’s Note

All claims expressed in this article are solely those of the authors and do not necessarily represent those of their affiliated organizations, or those of the publisher, the editors and the reviewers. Any product that may be evaluated in this article, or claim that may be made by its manufacturer, is not guaranteed or endorsed by the publisher.
