# Supplementary material for: Dynamics of T-Lymphocyte Activation Related to Paradoxical Tuberculosis-Associated Immune Reconstitution Inflammatory Syndrome in Persons With Advanced HIV
Source: Front Immunol. 2021 Oct 7;12:757843. doi: 10.3389/fimmu.2021.757843 (PMC8529328; doi:10.3389/fimmu.2021.757843)
Supplement: Supplementary file 1 [file DataSheet_1.pdf]

Dynamics of T-lymphocyte activation related to paradoxical tuberculosis-associated immune reconstitution inflammatory syndrome in persons with advanced HIV

Rafael Tibúrcio, Beatriz Barreto-Duarte, Narendran Gopalan, Artur T. L. Queiroz, Selvaraj Anbalagan, Kaustuv Nayak, Narayanan Ravichandran, Rajasekaran Subramani, Lis R. V. Antonelli, Kumar Satagopan, Komathi Anbalagan, Brian O. Porter, Alan Sher,Soumya Swaminathan, Irini Sereti and Bruno B. Andrade

Supplementary Material

Content

1. Supplementary Table 1

Supplementary Table:

Supplementary Table 1 - Characteristics of the study participants

**Patient characteristics**	**Non-IRIS (N = 25)**	**TB-IRIS (N = 31)**	**p-value**
Male Gender - n (%)	20 (80%)	18 (58%)	0.735
Median Age - years (IQR)	37 (31-40)	36 (27-46)	0.967
Median BMI - kg/m² (IQR)	40.5 (35.7-46.5)	42.0 (36.0-48.0)	0.732
Median Time to ART - days (IQR)	43 (23-68)	20 (14-30)	< 0.001
**Hematologic characteristics**			
$CD4^+$ T lymphocytes/µL – median (IQR)	156 (89-264)	93 (39- 135)	0.005
$CD8^+$ T lymphocytes/µL – median (IQR)	459 (297 -727)	765 (311 -1095)	0.109
HIV RNA copies – median $\log_{10}$/mL plasma (IQR)	5.3 (4.5 -5.6)	5.9 (5.4 – 5.9)	< 0.001

Table Note: Data represent no (%) of participants unless otherwise indicated. Data were compared using the Mann Whitney U test (continuous variables) or the Fisher's exact test. IQR=interquartile range; BMI=body mass index; ART=antiretroviral therapy
